# Development of Root Caries Associated With the Use of Sugar-Free Nicotine Lozenges: A Long-Term Case Report

**DOI:** 10.1155/2024/6635130

**Published:** 2024-08-07

**Authors:** Coral Ehrhardt, Kauko K. Mäkinen, Charles M. Cobb

**Affiliations:** ^1^ Moellers & Moellers Family Dentistry, 511 W Water St, Suite C, Decorah, Iowa 52101, USA; ^2^ Institute of Dentistry University of Turku, FI-20014 Turun yliopisto, Turku, Finland; ^3^ School of Dentistry The University of Michigan, 1011 N University Ave, Ann Arbor, Michigan 48104, USA; ^4^ Department of Periodontics School of Dentistry University of Missouri-Kansas City, 650 E. 25th Street, Kansas City, Missouri 64108, USA

**Keywords:** dental caries, lozenge, maltodextrin, mannitol, root caries, sugar alcohols, tobacco cessation aids

## Abstract

The authors present a case concerning an adult male patient who developed multiple sites of root caries adjacent to the area where he habitually held a sugar-free nicotine lozenge that contained mannitol and maltodextrin. The root caries occurred despite the patient's excellent oral hygiene, exemplary dietary habits, and clinically normal salivary flow. Between 1999 and 2008, he had only required two restorations to repair carious lesions. This patient had a 20+-year habit of using smokeless tobacco before switching to a cessation aid nicotine lozenge in May of 2008. A full-mouth series of radiographs taken in November 2009 revealed carious lesions on virtually every posterior tooth. The nicotine lozenge's principal ingredients were mannitol (75.7%) and maltodextrin. According to the United States' current Food and Drug Administration (FDA) guidelines, manufacturers can advertise these lozenges as sugar-free. Thus, it is assumed by the public that these types of products are incapable of “causing a cavity.” However, this case report presents evidence suggesting that frequent use of sugar-free nicotine lozenges may be associated with dental caries.

## 1. Introduction

Most consumers and even dental professionals regard “sugar-free” products as dentally harmless. They reasonably assume that a “sugar-free” label means a product is devoid of sugar. However, in the United States, products can legally contain up to 0.5 g of sugars per serving and still be labelled “sugar-free” [1]. This definition of “sugar-free” may be sufficient for food items, but there is no special consideration for small items such as a lozenge, cough drop, or hard candy, where a half gram of sucrose would represent a significant percentage of the product. Furthermore, all these types of products are designed to slowly melt in the mouth instead of being swallowed. This means longer exposures to oral bacteria and salivary amylase [2], thereby allowing more fermentation to occur. Even small amounts of sugar could have a cariogenic potential with long and frequent use.

The nicotine lozenges used by the patient in this case report were initially suspected of containing the maximum legal level of sugars. However, professional food testing results indicated that it contained none. So why are these lozenges still suspected of causing rampant root caries is because they contain other fermentable ingredients besides sucrose.

Slow-dissolving products like medicated lozenges, cough drops, and hard candies require bulking agents for structure (literally, something to suck on). Our case report patient's lozenge contained mannitol (75.7%), a low-calorie sugar alcohol of the hexitol type, which is known to be fermentable by oral bacteria. According to the package label, this nicotine lozenge also contained maltodextrin, a polysaccharide. Given the time, the enzymes in human saliva can break down maltodextrin into simple sugars [2]. While neither of these ingredients are technically “sugars,” they are both carbohydrate in nature and under suitable conditions and can produce acids in the oral environment [3].

**Figure 1:**
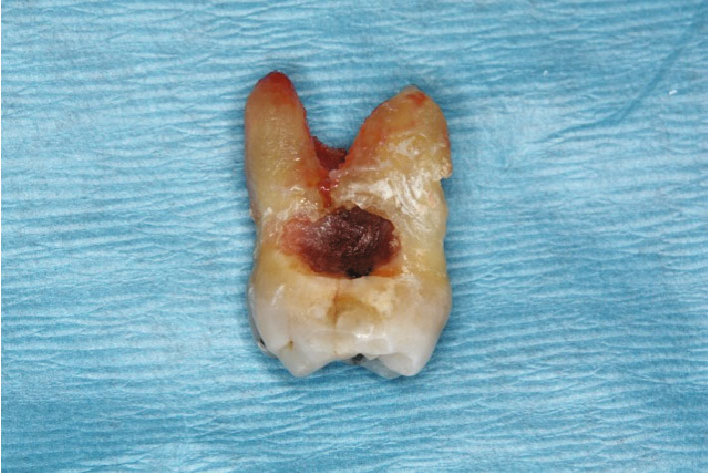
Maxillary second molar, FDI 17 (US #2) showing extensive mesial surface root caries.

Our case report patient was unaware of tooth decay issues potentially associated with his use of a nicotine lozenges advertised as “sugar-free.” In fact, he assumed it was a healthier habit than returning to his smokeless tobacco habit. Unfortunately, within 19 months of usage, this patient developed carious lesions on 10 of his posterior teeth. Tooth FDI 17 (US #2) was deemed unrestorable and extracted (Figure 1). The Food and Drug Administration (FDA) considers mannitol to be a “tooth-friendly” sweetener [1] because it produces less acid when fermented than does sucrose. However, this might be an oversimplified and incorrect assumption. This case study calls into question the cariogenic potential of nicotine lozenges containing mannitol and maltodextrin and more broadly questions the dental safety of current FDA standards for labelling these types of sugar-free products.

## 2. Case Presentation

### 2.1. Medical History

In 2009, the patient was a healthy 58-year-old male and under the care of a medical doctor. He reported no significant medical issues or use of prescription medication. This patient abstained from alcohol and/or recreational drugs. His only medical concern was a 20+-year habit of using smokeless tobacco after he quit smoking in the 1980s. At a prophylaxis appointment in June 2008, he reported quitting smokeless tobacco 6 weeks earlier. From 1991 to 2009, his medical history was updated at each appointment, both verbally and by a written questionnaire.

### 2.2. Dental and Radiographic History

**Figure 2:**
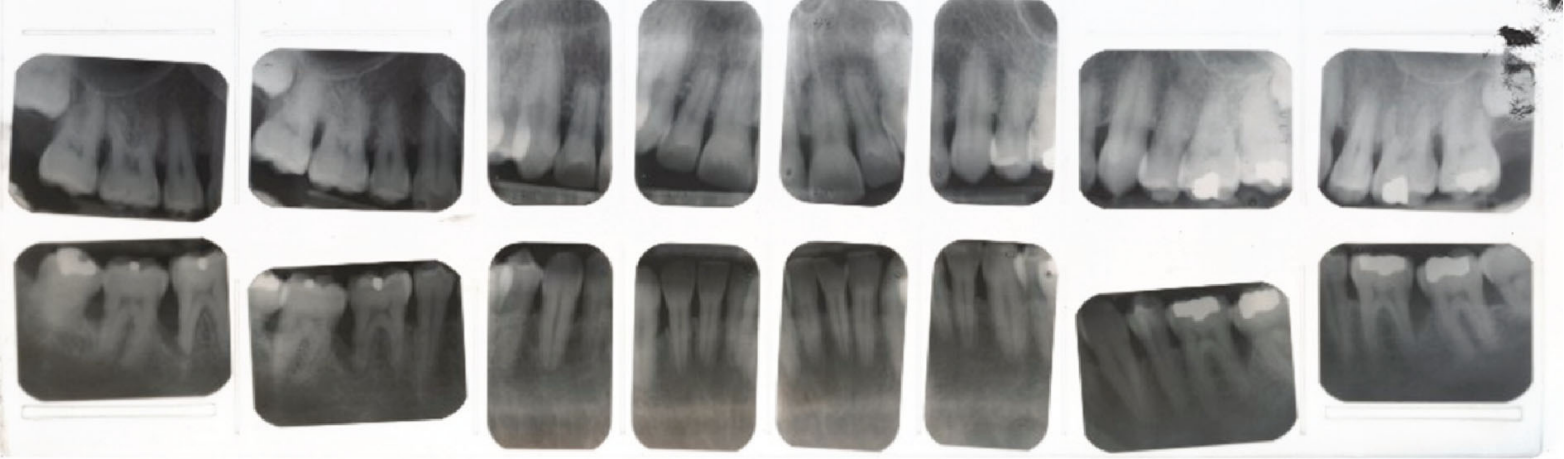
Copy of the original full-mouth radiographic survey (FMRS) taken at the periodontal office, January 9, 1991.

**Figure 3:**
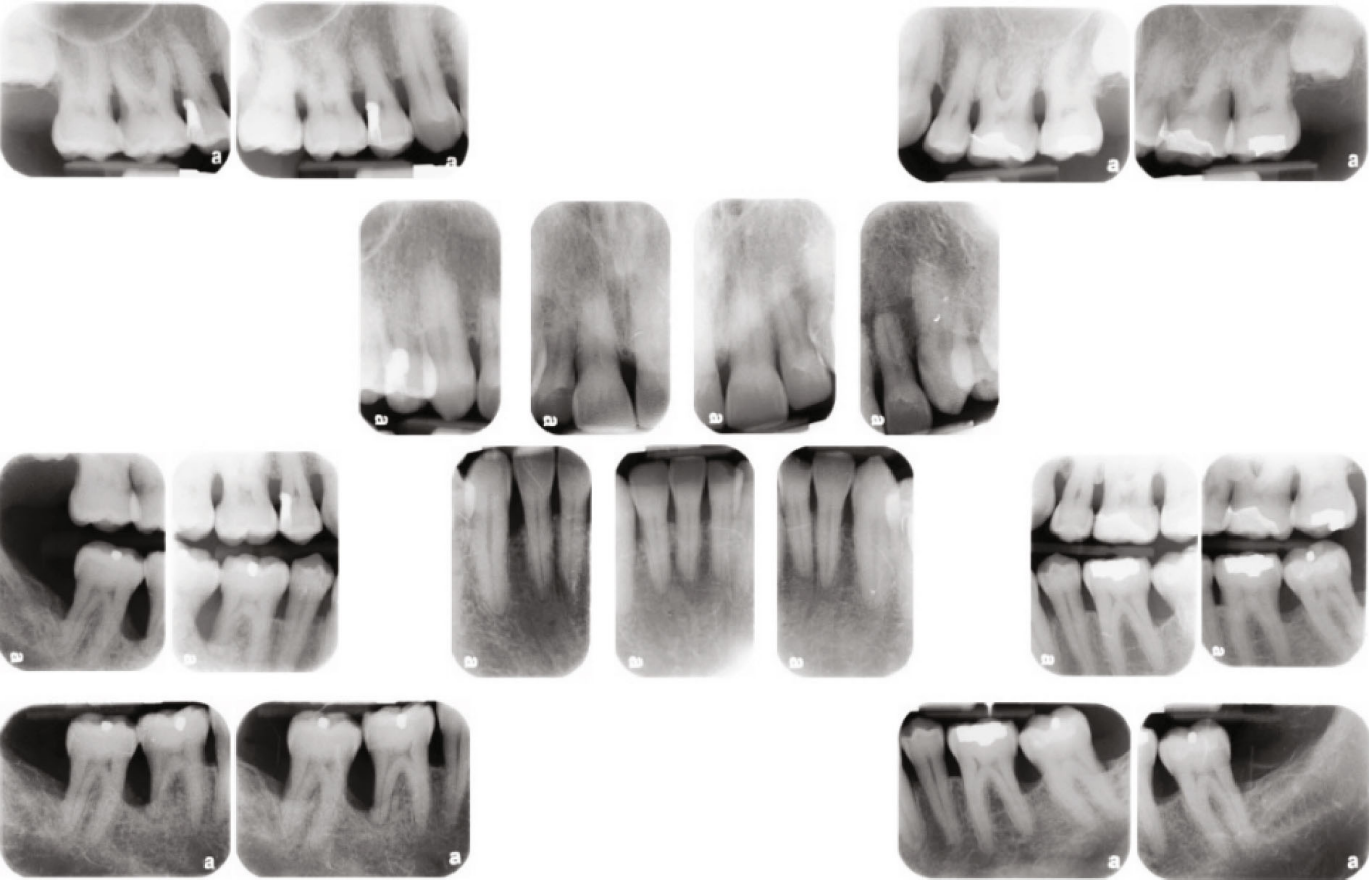
Full-mouth radiographic survey (FMRS) taken at the periodontal office, November 20, 2006.

This patient had been seen regularly in the office by the same general dentist since 1988. Between the years 1999 and 2008, he had required only two restorations due to caries, a distal occlusal on FDI 15 (US #5) and an occlusal on FDI 37 (US #18). The treatment history indicated that his general dentist took vertical bitewing radiographs every 12–18 months. In addition, a full-mouth radiographic survey (FMRS) was taken every 3–5 years at a periodontal office (Figures 2 and 3).

### 2.3. Periodontal and Oral Hygiene History

In December 1990, the patient was referred by his general dentist to a periodontist for treatment of Stage III, Grade B periodontitis [4]. The patient started periodontal treatment in January 1991, consisting of full-mouth scaling and root planing and reevaluation of the response to therapy. Starting in October 1991, periodontal surgery was performed, consisting of mucoperiosteal flap reflection for access, root planing, osteoplasty, and apical positioning to facilitate reduction in probing depths. After healing, the patient was placed on a 3-month periodontal maintenance schedule, alternating with the general dentist. Further surgical intervention, consisting of osseous grafting, was done in March 1991. The patient again alternated periodontal maintenance appointments with good compliance through November 2009.

At each periodontal maintenance appointment, since 1992, disclosing solution was used, plaque indexes were recorded, and oral hygiene was reviewed. The patients' plaque index assessments were consistently low. His daily oral hygiene practices included use of both floss and interproximal brushes. The patient typically presented with very little to no gingival inflammation. From a dietary standpoint, he did not consume soda or any other sugary beverages. He rarely consumed sweets and disclosed his only “vice” had been using smokeless tobacco.

### 2.4. Dental Status at Diagnosis

In June 2008, the patient was seen by his general dentist for four vertical bitewings, exam, and cleaning. The notes indicated the patient reported quitting smokeless tobacco 6 weeks earlier. One lesion of root caries was detected on the distal of tooth FDI 16 (US #3) and repaired several weeks later with a dual-cure, self-adhesive glass ionomer. At his November 2009 periodontal maintenance appointment, soft tooth structure was clinically detected on the mesial of tooth FDI 17 (US #2). The patient was also experiencing sensitivity on the upper right. A periapical film was taken to screen for endodontic involvement and confirm the presence of caries. No endodontic lesion was radiographically evident, but the location and the severity of the root caries rendered the tooth unrestorable, resulting in extraction (Figure 1).

**Figure 4:**
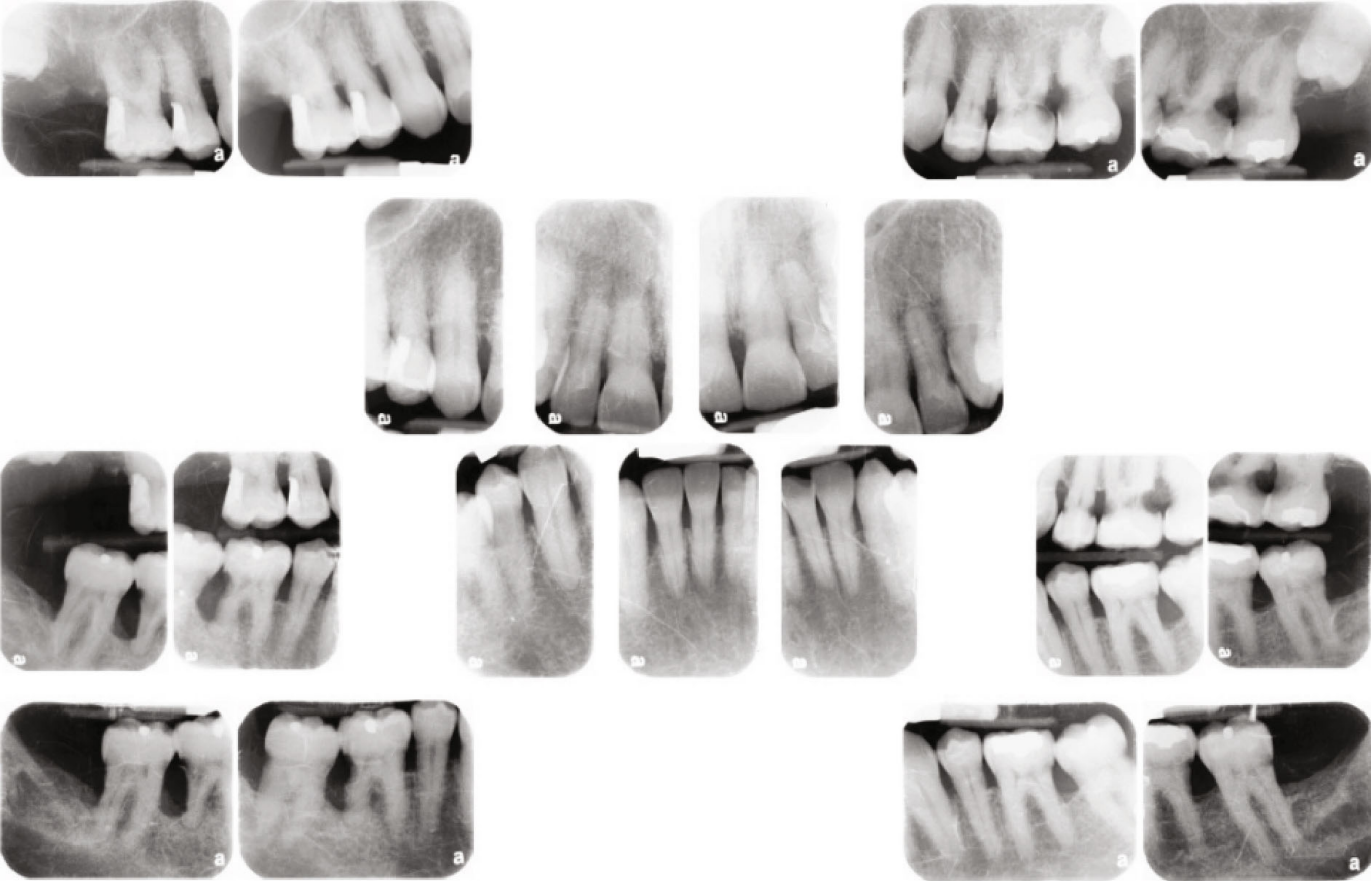
Full-mouth radiographic survey (FMRS) taken at the periodontal office, December 1, 2009.

The patient was seen the following week for a FMRS and examination by the periodontist. There was radiographic evidence of root caries on FDI 24, 26, 28, 37, 36, 46, and 47 (US #12, #14, #15, #18, #19, #30, and #31) (Figure 4).

**Figure 5:**
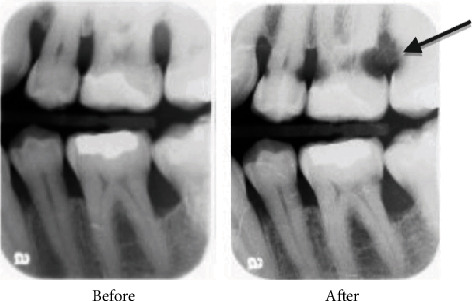
Vertical bitewing radiographs of the left maxillary and mandibular first molars. The “before” was taken November 20, 2006. The “after” was taken December 1, 2009, 19 months after the patient started using nicotine lozenges. Note the extensive root caries involving the mesial and distal surfaces of the maxillary teeth and distal of the mandibular first molar.

All previous FMRSs indicated a stable periodontium and no evidence of enamel or root surface caries. The patient had also disclosed “quitting chew” in 2008 to the periodontal office but was more forthcoming about his continued reliance on an over-the-counter (OTC), sugar-free nicotine lozenge. This prompted further questioning about the lozenge or changes in dietary habits and/or medical issues. No significant adjunctive or contributory factors were discovered that would promote a sudden and rapid carious process, except possibly the nicotine lozenges. A side-by-side comparison of vertical bitewings highlights the startling change (Figure 5).

### 2.5. Medicated Nicotine Lozenges and Laboratory Findings

Nicotine oral lozenges are intended to aid tobacco cessation by lessening withdrawal effects. They replace the nicotine one would receive from tobacco with nicotine in a medicated lozenge. Nicotine, IUPAC name 3-[(2S)-1-methylpyrrolidin-2-yl]pyridine (CAS Number 544-11-5), is slowly released from the lozenge and enters the bloodstream through the oral mucosal tissues [5]. The brand used by our case report patient was available OTC and came in several palatable flavors, including mint, cherry, and cappuccino. According to the manufacturers' instructions (shown on the package and insert), the lozenges were designed to be “parked” in the cheek. Each lozenge takes at least 20–30 min to completely dissolve and leaves adjacent teeth coated with the substance for even longer. The manufacturer instructed consumers to begin using at least 9 lozenges a day but could “safely use up to 20 lozenges” daily.

In the United States, OTC products like medicated lozenges and cough drops have their ingredients listed in alphabetical order. Unfortunately, this labelling system gives no indication of the percentage or predominance of any particular ingredient. Consequently, the author enlisted Anresco Laboratories (San Francisco, CA) for an analysis of sugar content. Anresco is an accredited laboratory for analytical testing services for food-related industries and recognized by the US FDA. The cappuccino nicotine lozenge was submitted for testing because it was the flavor most used by the patient. The analysis revealed the lozenge contained no sugars, including fructose, glucose, sucrose, and maltose. This same lab also identified the main ingredient, mannitol (75.7%) [6]. Mannitol is a common sugar alcohol currently classified as a “tooth-friendly” and “noncariogenic” sweetener by the FDA [1]. Based on common lozenge formularies, it is estimated that the content of maltodextrin is approximately 20%. Anresco laboratory also reported that the lozenge had a pH of 9.37.

### 2.6. Treatment Outcomes

As discussed earlier, in June 2008 (approximately 6 weeks after he began using the sugar-free nicotine lozenge), vertical bitewings were taken at his general dentist's office during a routine exam and prophylaxis appointment. One area of root caries was detected clinically, not radiographically, on the mesial of FDI 16 (US #3) and repaired with a glass ionomer 2 weeks later. Coincidentally, this new carious activity was directly adjacent to one of his preferred spots to hold the nicotine lozenge while it dissolved.

When a FMRS and a single periapical film were taken 19 months later at the periodontal office, those radiographs revealed multiple areas of root caries. Two of his maxillary molars, FDI 17 and 27 (US #2, #15), were deemed unrestorable and extracted. FDI 26 (US #14) was endodontically treated and restored with a porcelain fused to metal crown.

**Figure 6:**
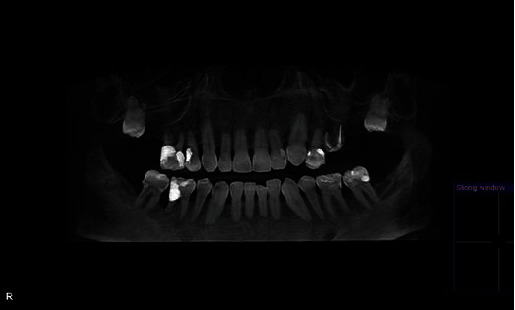
Panoramic radiograph (circa 2016) taken before extractions.

**Figure 7:**
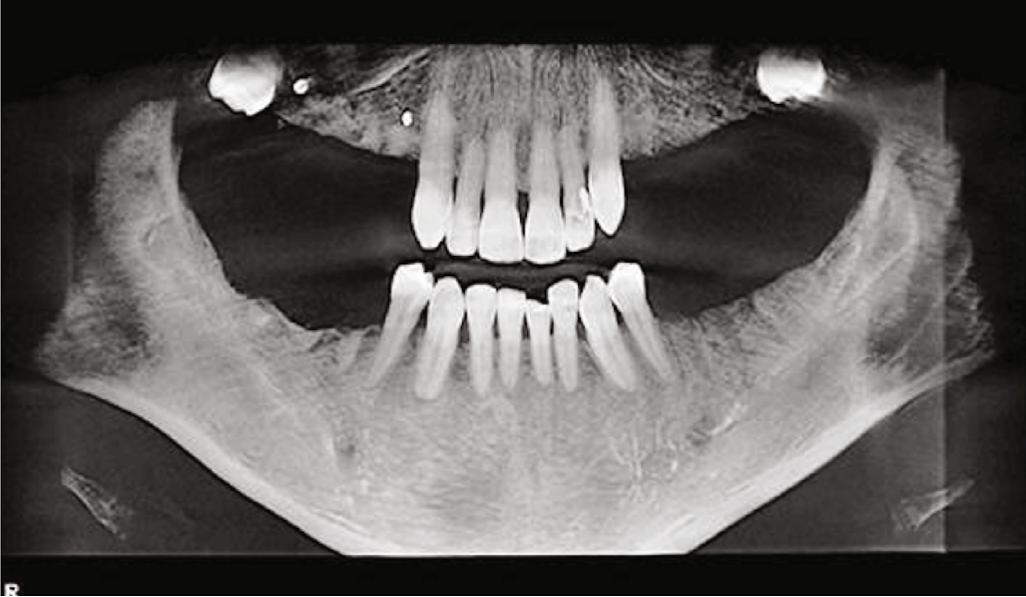
Panoramic radiograph (circa 2016) following extraction of all posterior teeth, except mandibular first bicuspids. Impacted third molars were not removed due to distinct possibility of ankylosis and potential for fracturing the posterior wall and floor of the maxillary antrum.

**Figure 8:**
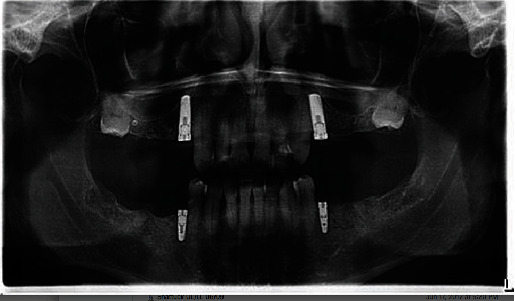
Panoramic radiograph (March 2017) after placement of dental implants and treatment planned for a shortened dental arch.

Caries lesions were also radiographically detectable on FDI 24, 36, 37, 46, and 47 (US #12, #18, #19, #30, and #31). Those teeth were promptly restored with glass ionomer, but due to the sudden and aggressive nature of the caries, all were given guarded prognoses. As feared, all restorations placed between December 2009 and March 2010 developed recurrent caries. Over the next 5 years, all these restorations were either replaced or “patched” at least once (Figure 6). By 2016, the general dentist and patient decided the best course of action was to extract all remaining molars and premolars, except tooth FDI 34 and 44 (US #21, #28) (Figure 7). In March of 2017, he had four implants placed and treatment planned for a shortened dental arch (Figure 8).

Regrettably, this case report patient continues to habitually use one of the many generic nicotine lozenges available OTC today. Fortunately, several mitigating changes have occurred. First, the nicotine lozenge he now uses is much smaller (approximately half the size) and dissolves more quickly. Second, it contains no maltodextrin, although it still contains mannitol. And finally, the patient attempts to isolate the lozenge by holding it against his palate with his tongue. Unfortunately, even with all these modifications to usage, caries continues to be an issue for this patient.

**Figure 9:**
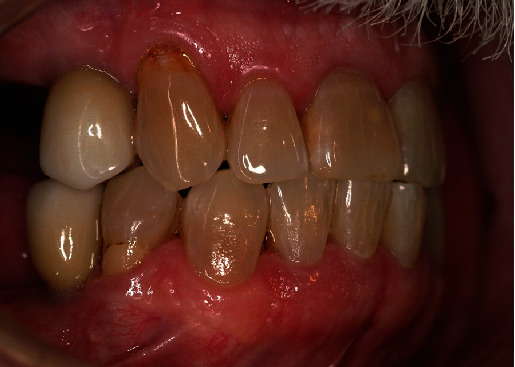
Case report patient, June 21, 2024.

Between June 2020 and October 2022, he required Class V fillings on FDI 13, 22, and 44 (US #6, #10, and #28). These lesions occurred on the facial and buccal surfaces despite his new habit of holding the lozenge on his palate. We believe this is happening for two reasons. First, we suspect that enough dissolved substrate still contacts his entire dentition by seeping through the edentulous and interproximal areas. Second, the areas that developed carious lesions are also the only areas with gingival recession and exposed roots. It appears his enamel can withstand the slight but steady drop in pH while the more susceptible roots are succumbing to demineralization. At his last dental exam in June of 2024, tooth FDI 13 (US #6) was suspected of having recurrent root caries (Figure 9).

## 3. Discussion

The sugar-free nicotine lozenge used by the patient in this case report contained mannitol (75.7%) and maltodextrin. Acid production from mannitol by dental plaque or by specific oral microorganisms can be considered significantly less common than that from related aldoses or disaccharides (such as glucose, sucrose, and maltose). The scientific literature is replete with reports of acid production from mannitol and with findings suggesting the involvement of mannitol-specific phosphotransferase systems in *Streptococcus mutans* [7–10].

Examples of acid formation from mannitol in dental plaque, or in laboratory experiments, include those involving *Lactobacillus* strains [11, 12], *S. mutans* [13–15], and *Streptococcus sobrinus* [16, 17]. Shemesh et al. [14] specifically pointed out that although sorbitol and mannitol are considered noncariogenic sugar substitutes, they may indirectly affect caries by promoting biofilm formation via enhanced expression of glucosyltransferase and fructosyltransferase systems. Earlier literature reviews support these suggestions and, de facto, predicted the advent of the abovementioned findings on specific microorganisms [18, 19].

The lozenge in this report was comprised of 75.7% mannitol, but it also contained maltodextrin, a carbohydrate filler often used to increase texture and mouthfeel. When maltodextrin is dissolved by saliva, it can leave a film on tooth surfaces. Maltodextrin solutions are less acidogenic than corresponding sucrose solutions; nevertheless, it can affect tooth enamel [20]. Maltodextrin is a polysaccharide consisting of D-glucose units connected in carbohydrate chains of variable length, the units being primarily linked with *α*(1→4) glycosidic bonds [20]. Typical maltodextrin molecules may comprise 3–17 glucose units. These glycosidic bonds can be hydrolyzed in oral conditions by microbial and salivary amylase and other glucosidase-type enzymes. Hydrolysis results in the formation of monosaccharides (mainly glucose molecules) which are subject to fermentation and acid formation.

It would seem several things contributed to this sugar-free nicotine lozenge causing rampant root surface caries for this case report patient. First and foremost, two of the main ingredients (mannitol and maltodextrin) are fermentable by oral bacteria. Granted, they both produce less acids than sucrose, but the exposure times were extremely long and localized. The instructions advised consumers to “park” the lozenge while it slowly dissolved. This concentrated the fermentable ingredients in one area and impaired the saliva's ability to neutralize acids. Second, clearance of these substrates was poor, with maltodextrin likely helping to create a sticky matrix. This fermentable film would cover the adjacent teeth and their roots for a minimum of 30 min per lozenge. And finally, the patient used these nicotine lozenges 9–20 a day for well over a year. Since the product was labelled sugar-free and there was no warning on the package about possible tooth decay, this patient assumed that using a nicotine lozenge was safer than chewing tobacco.

Having addressed the potential role for sugar-free nicotine lozenges in dental root caries, three confounders must be considered as they may influence the potential for root caries. The first confounder is the role of xerostomia. It is well-known that the prevalence of xerostomia increases with increasing age, use of specific medications (i.e., drug induced xerostomia), and following radiation treatment [21, 22]. It is also well established that the incidence of enamel and root caries increases in the presence of xerostomia [23, 24]. The patient in this case did not have any indication of xerostomia. However, xerostomia should always be evaluated as a risk factor for increased caries incidence in older patients using OTC nicotine troches.

The second confounder is the role of exposed root surfaces. This case report patient's gingival recession was significant in the posterior regions due to the loss of attachment from periodontal disease and/or resultant surgical intervention. At least one study reports that increasing age, gender, and a higher number of retained teeth are significant risk indicators for root caries [25]. Interestingly, the literature is somewhat conflicted regarding the prevalence of root caries in patients treated for periodontal disease [26]. Li et al. [27] using meta-analysis determined that periodontitis patients had an odds ratio (OR) of 2.10 (95% CI: 1.03–4.29). Despite an OR of 2.1, Keltjens et al. [28] reported a root caries index score of only 6.28% in patients treated for periodontal disease. The authors also noted that posterior teeth were more affected than were anterior teeth. Similar results were reported by Ravald and Hamp [29] in that less than 5% of exposed root surface exhibited root caries in patients treated for advanced periodontal disease.

The third confounder is the role of smokeless tobacco. The literature shows tobacco usage to be associated with oral cancer, various mucosal lesions, periodontal disease, and enamel caries [30]. Somewhat surprisingly, there are few reported studies looking at tobacco use of any type and associated root caries [30–32]. At least one report concluded that patients using tobacco products experience significantly more root caries than nonsmokers [26]. Gavriilidou and Belibasakis [33] summed the evidence stating the epidemiological and pathophysiological features of root caries and influencing factors such as aging, xerostomia, medications, and changes in the oral ecology and microbiome as periodontal disease may function synergistically to increase the risk for root caries.

## 4. Conclusions

This case study presents clinical evidence that frequent use of a sugar-free nicotine lozenge was associated with the development of rampant root caries. This occurred in spite of the patient being a healthy adult with normal salivary flow and good oral hygiene habits. The implication of this finding can be extrapolated to other products assumed to be dentally harmless such as sugar-free cough drops and hard candies.

The authors acknowledge that this case report patient's usage of the nicotine lozenge was extreme. However, dental professionals know from clinical experience that sugar-free products are sometimes habitually used by patients suffering from xerostomia to relieve the discomforts of a dry mouth. Dentists and hygienists also understand that exposure times and frequency of use play a major role in determining the cariogenicity of a substrate, not just levels of sweetness or sucrose content. For example, nursing bottle caries studies show that even mother's milk is capable of causing tooth decay if exposures are excessive [34, 35].

Slow-dissolving products, like lozenges and cough drops, are intended to be held in the mouth for long periods of time instead of being swallowed. The sugar-free nicotine lozenge in this report contained maltodextrin, a polysaccharide that is derived from either corn, rice, potato starch, or wheat. These longer exposures in the mouth allow amylase, an enzyme in saliva, to break down carbohydrates to simpler sugars [2]. Long exposures also mean that “tooth-friendly” ingredients with low fermentation rates (i.e., mannitol and sorbitol) have exponentially longer times to ferment, thus producing more acids.

Current FDA guidelines allow small, slow-melting lozenges to be labelled “sugar-free” if they contain less than 0.5 g of sugars. They can also be labelled “calorie-free” if they contain less than 5 calories per serving [1]. For comparison, regular sucrose base cough drops have approximately 10–15 calories per serving. Labeling products as “sugar-free” and “calorie-free” implies a product is completely innocuous and may even encourage overuse. Ideally, FDA labeling guidelines should be modified and more research should be done on this issue. Until then, dental professionals should caution patients (particularly those with xerostomia and root exposure) about potential caries risks associated with sugar-free products.

## Data Availability

The data is available from the corresponding author upon request.

## References

[1] Bobka M. S. (1993). The 21 CFR (Code of Federal Regulations) online database: Food and Drug Administration regulations full-text. *Medical Reference Services Quarterly*.

[2] Gachons C. P. D., Breslin P. A. (2016). Salivary amylase: digestion and metabolic syndrome. *Current Diabetes Reports*.

[3] Holesh J. E., Aslam S., Martin A. (2024). Physiology, Carbohydrates. *StatPearls*.

[4] Tonetti M. S., Greenwell H., Kornman K. S. (2018). Staging and grading of periodontitis: framework and proposal of a new classification and case definition. *Journal of Periodontology*.

[5] Benowitz N. L., Jacob P., Jones R. T., Rosenberg J. (1982). Interindividual variability in the metabolism and cardiovascular effects of nicotine in man. *The Journal of Pharmacology and Experimental Therapeutics*.

[6] Kushi C. (2011). *File No. 921106324*.

[7] Maryanski J. H., Wittenberger C. L. (1975). Mannitol transport in Streptococcus mutans. *Journal of Bacteriology*.

[8] Honeyman A. L., Curtiss R. (1992). Isolation, characterization, and nucleotide sequence of the Streptococcus mutans mannitol-phosphate dehydrogenase gene and the mannitol-specific factor III gene of the phosphoenolpyruvate phosphotransferase system. *Infection and Immunity*.

[9] Honeyman A. L., Curtiss R. (2000). The mannitol-specific enzyme II (mtlA) gene and the mtlR gene of the PTS of Streptococcus mutans. *Microbiology (Reading)*.

[10] Gong T., He X., Chen J. (2021). Transcriptional profiling reveals the importance of RcrR in the regulation of Multiple sugar transportation and biofilm formation in streptococcus mutans. *mSystems*.

[11] Liong M. T., Shah N. P. (2005). Production of organic acids from fermentation of mannitol, fructooligosaccharide and inulin by a cholesterol removing Lactobacillus acidophilus strain. *Journal of Applied Microbiology*.

[12] Almstuhl A., Lingström P., Eliasson L., Carlén A. (2013). Fermentation of sugars and sugar alcohols by plaque Lactobacillus strains. *Clinical Oral Investigations*.

[13] Stösser L., Rohland F., Seyfarth W. (1989). The spectrum of S. mutans OMZ 176 products from different mono- and disaccharides. *Zahn-, Mund-, und Kieferheilkunde mit Zentralblatt*.

[14] Shemesh M., Tam A., Feldman M., Steinberg D. (2006). Differential expression profiles of Streptococcus mutans ftf, gtf and vicR genes in the presence of dietary carbohydrates at early and late exponential growth phases. *Carbohydrate Research*.

[15] Ajdić D., Pham V. T. (2007). Global transcriptional analysis of Streptococcus mutans sugar transporters using microarrays. *Journal of Bacteriology*.

[16] Gehring F., Einwag J. (1990). Demonstration of Streptococcus sobrinus in human oral cavity. *Oral-Prophylaxe*.

[17] Ma Y., Lassiter M. O., Banas J. A., Galperín M. Y., Taylor K. G., Doyle R. J. (1996). Multiple glucan-binding proteins of Streptococcus sobrinus. *Journal of Bacteriology*.

[18] Toors F. A. (1992). Chewing gum and dental health. Literature review. *Revue Belge de Medecine Dentaire*.

[19] Edgar W. M., Dodds M. W. (1985). The effect of sweeteners on acid production in plaque. *International Dental Journal*.

[20] Hofman D. L., van Buul V. J., Brouns F. J. (2016). Nutrition, health, and regulatory aspects of digestible maltodextrins. *Critical Reviews in Food Science and Nutrition*.

[21] Guggenheimer J., Moore P. A. (2023). Xerostomia: etiology, recognition and treatment. *Journal of the American Dental Association*.

[22] Felix D. H., Luker J., Scully C. (2012). Oral Medicie: 4. Dry mouth and disorders of salivation. *Dental Update*.

[23] de Mata C., McKenna G., Burke F. M. (2011). Caries and the older patient. *Dental Update*.

[24] Youngs G. (1994). Risk factors for and the prevention of root caries in older adults. *Special Care in Dentistry*.

[25] Reddy L. S., Lakshmi S. V., Lakshmi Y. V., Lakshmi P. D., Sravanthi Y., Kaur M. (2021). Root caries experience and its association with risk indicators among middle-aged adults. *Journal of Pharmacy & Bioallied Sciences*.

[26] Ravald N., Birkhed D., Hamp S. E. (1993). Root caries susceptibility in periodontally treated patients. *Journal of Clinical Periodontology*.

[27] Li Y., Xiang Y., Ren H. (2024). Association between periodontitis and dental caries: a systematic review and meta-analysis. *Clinical Oral Investigations*.

[28] Keltjens H., Schaeken T., van der Hoeven H., Hendriks J. (1988). Epidemiology of root surface caries in patients treated for periodontal diseases. *Community Dentistry and Oral Epidemiology*.

[29] Ravald N., Hamp S. E. (1981). Prediction of root surface caries in patients treated for advanced periodontal disease. *Journal of Clinical Periodontology*.

[30] Gajendra S., McIntosh S., Ghosh S. (2023). Effects of tobacco product use on oral health and the role of oral healthcare providers in cessation: a narrative review. *Tobacco Induced Diseases*.

[31] Xue J., Jiang X., Wang Y., Huang R. (2019). Correlation between tobacco smoking and dental caries: a systematic review and meta-analysis. *Tobacco Induced Diseases*.

[32] Aguilar-Zinser V., Irigoyen M. E., Rivera G., Maupomé G., Sánchez-Pérez L., Velázquez C. (2008). Cigarette smoking and dental caries among professional truck drivers in Mexico. *Caries Research*.

[33] Gavriilidou N. N., Belibasakis G. N. (2019). Root caries: the intersection between periodontal disease and dental caries in the course of ageing. *British Dental Journal*.

[34] Mathur V. P., Dhillon J. K. (2018). Dental caries: a disease which needs attention. *Indian Journal of Pediatrics*.

[35] Bowen W. H., Lawrence R. A. (2005). Comparison of the cariogenicity of cola, honey, cow milk, human milk, and sucrose. *Pediatrics*.

